# Investigating the effect of the South African Adolescence Sleep Intervention (SAASI) on adolescent sleep and PTSD: A pilot randomized control study

**DOI:** 10.1192/j.eurpsy.2025.1966

**Published:** 2025-08-26

**Authors:** S. Suliman

**Affiliations:** 1 South African Medical Council Unit on the Genomics of Brain Disorders, and Department of Psychiatry, Stellenbosch University, Cape Town, South Africa

## Abstract

**Introduction:**

South African adolescents are exposed to significant levels of trauma exposure, resulting in high levels post-traumatic stress disorder (PTSD). Sleep disturbances are among the most frequently reported difficulties faced by those dealing with PTSD.

**Objectives:**

The current study aimed to determine the feasibility and preliminary efficacy of the SAASI on PTSD symptom severity and sleep disturbance when delivered in group format to South African adolescents with PTSD.

**Methods:**

Sixty-one adolescents with PTSD diagnoses and sleep disturbance were randomly assigned to either one individual and four group sessions of the sleep intervention (SAASI) or a control group. At baseline, post- and 1-month follow-up participants completed the Child PTSD symptom scale for DSM5 (CPSS-5) and the Pittsburgh Sleep Quality Index (PSQI) among other sleep and psychiatric measures. The trial was registered on the Pan African Trial Registry (PACTR202208559723690).

**Results:**

There was a significant but similar decrease in PSQI scores in both groups over time indicating no overall intervention effect. Interaction between groups on the CPSS-5 was also not significant. Despite this overall finding, the mean difference in CPSS-SR-5 scores increased over time, with the difference between groups post-treatment and at the 1-month follow-up suggesting that PTSD symptom severity decreased more in the intervention group than the control group. The dropout rate was higher than expected for both the intervention and control groups. Reasons provided for dropout were mostly school commitments or travel related.

**Conclusions:**

Conclusions: Early findings suggest a trend towards dual improvement in sleep quality and PTSD symptom severity in adolescents with a sleep disturbance and PTSD receiving a group sleep intervention (SAASI). Further investigation in a properly powered RCT with detailed retention planning is indicated.

**Disclosure of Interest:**

None Declared

